# Updates on the Prevalence, Quality of Life, and Management of Chronic Cough in Interstitial Lung Diseases

**DOI:** 10.3390/diagnostics15091139

**Published:** 2025-04-29

**Authors:** Natalia V. Trushenko, Olga A. Suvorova, Anna E. Schmidt, Svetlana Y. Chikina, Iuliia A. Levina, Baina B. Lavginova, Sergey N. Avdeev

**Affiliations:** 1 Pulmonology Department, Sechenov First Moscow State Medical University (Sechenov University), Healthcare Ministry of Russia, Trubetskaya St. 8, Build. 2, Moscow 119991, Russia; 2 Pulmonology Scientific Research Institute, Federal Medical and Biological Agency of Russia, Orekhovyy Boulevard 28, Moscow 115682, Russia

**Keywords:** chronic cough, interstitial lung disease, idiopathic pulmonary fibrosis, sarcoidosis, cough pathophysiology, health-related quality of life, cough treatment, P2X3 receptor antagonists

## Abstract

**Background:** Chronic cough is a common symptom in patients with interstitial lung diseases (ILDs), which significantly affects health-related quality of life (HRQoL). The prevalence of chronic cough varies from 30% to almost 90% in different ILDs, with the highest rate in patients with idiopathic pulmonary fibrosis. However, the pathophysiology of cough in ILDs remains poorly understood, with multiple proposed mechanisms contributing to its development. This knowledge gap complicates both clinical assessment and treatment, as current therapeutic strategies target general cough mechanisms rather than ILD-specific pathways. This review synthesizes existing data to clarify distinct cough mechanisms across ILD subtypes and identify opportunities for more targeted therapeutic strategies in this challenging patient population. Moreover, cough can be a clinical marker of disease severity and a predictor of ILD progression and transplant-free survival. Effective cough-specific therapeutic options that consider potential mechanisms, comorbidities, and individual effects on HRQoL are needed for cough associated with ILD. Therefore, the aim of this review was to analyze the prevalence, the impact on HRQoL, the pathophysiology, and the management of chronic cough in ILDs. **Methods:** We performed a comprehensive search in PubMed, MEDLINE, Embase, and the Cochrane Library. This review included randomized clinical trials, observational studies, systematic reviews, and meta-analyses in adults with chronic cough comparing ILD types. The following were excluded: commentaries, letters, case reports and case series, conference abstracts, and studies and publications lacking cough-specific outcomes. **Results:** Several approaches to reduce cough frequency and severity were described: antifibrotic agents, neuromodulators, opiates, inhaled local anesthetics, oxygen, speech therapy, and anti-reflux therapy. Some therapeutic approaches, such as oral corticosteroids and thalidomide, can cause significant side effects. Novel agents, such as P2X3 receptor antagonists, which are in phase III trials (COUGH-1/2), show promising results for refractory cough and may benefit ILD-related cough. **Conclusions:** Thus, a comprehensive assessment of cough is required for effective cough treatment in patients with ILDs considering possible mechanisms and individual impact on QoL.

## 1. Introduction

Interstitial lung diseases (ILDs) are a heterogeneous group of diffuse diseases of the lung parenchyma, many of which are characterized by a progressive course and poor prognosis [1]. ILDs include idiopathic interstitial pneumonias (IIPs), hypersensitivity pneumonitis (HP), ILDs associated with connective tissue diseases (CTDs), sarcoidosis, and other ILDs (lymphangioleiomyomatosis, Langerhans cell histiocytosis, etc.).

There are limited and variable data on the prevalence of cough syndrome in patients with ILDs (Table 1). A number of studies have shown that about 83% of patients with idiopathic pulmonary fibrosis (IPF), 79% of patients with nonspecific interstitial pneumonia (NSIP), 84% of patients with HP, and 30–50% of patients with sarcoidosis suffer from cough [2,3,4,5,6]. The incidence of cough ranges from 32% to 75% in CTD-associated ILDs depending on the clinical variant of CTD [7].

Sato et al. showed that cough is 3.7-fold more frequent in patients with IIPs compared to patients with CTD-associated ILDs or HP (95% CI 1.7–8.2; *p* = 0.001) [8], while Cheng et al. demonstrated that the prevalence of cough was significantly higher in patients with IPF or HP compared to patients with systemic sclerosis-related ILD (SS-ILD) (87% and 83%, respectively, vs. 68%, *p* = 0.02) [9]. This variability in prevalence may reflect differences in diagnostic criteria and heterogeneity of the patient population and assessment methods, as there is no single validated approach to measuring cough in patients with ILDs.

**Table 1:** Prevalence of chronic cough in ILDs.

Disease	Prevalence	Reference
IPF	83.3% reported CC	Öz et al., ILD patients (total *n* = 69), 24 patients with IPF, 2022 [2]
	79.1% reported CC	Chikina et al., IPF patients (*n* = 1353), 2023 [5]
	87% reported CC	Cheng et al., total *n* = 176, 77 patients with IPF, 2017 [9]
HP	92.1% reported CC	Wang et al., HP patients (*n* = 101), 2019 [4]
	83% reported CC	Cheng et al., total *n* = 176, 32 with chronic HP, 2017 [9]
	3.7-fold more frequent in patients with IIPs compared to patients with CTD-ILDs or HP (95% CI 1.7–8.2; *p* = 0.001)	Sato et al., ILD patients (total *n* = 129), 10 patients with chronic HP, 70 patients with IIPs, 49 with CTD-ILDs, 2019 [8]
CTD-ILDs	56.2% reported CC	Öz et al., ILD patients (total *n* = 69), 32 patients with CTD-ILDs, 2022 [2]
	68% reported CC	Cheng et al., total *n* = 176, 67 patients with CTD-ILDs, 2017 [9]
	44.72% reported CC, ranging from 32.9% to 75% depending on the different CTD-ILDs	Lu et al., CTD-ILD patients (*n* = 161), 2022 [7]
IPAF	76.9% reported CC	Öz et al., ILD patients (total *n* = 69), 13 patients with IPAF, 2022 [2]
	41.67% reported CC	Lu et al., CTD-ILD patients (*n* = 161), 2022 [7]
Sarcoidosis	54.8% reported CC	Sinha et al., patients with sarcoidosis (*n* = 32), 2016 [6]

ILDs, interstitial lung diseases; IPF, idiopathic pulmonary fibrosis; CC, chronic cough; HP, hypersensitivity pneumonitis; IIP, idiopathic interstitial pneumonia; CTD-ILDs, connected tissue disease-associated interstitial lung diseases; IPAF, interstitial pneumonia with autoimmune features.

Chronic cough is one of the most common respiratory symptoms and significantly affects health-related quality of life (HRQoL) in patients with ILDs, which also serves as a benchmark for studying the effectiveness of cough therapy [10,11]. Additionally, chronic cough serves as an important clinical indicator in ILDs, demonstrating consistent associations with worsening pulmonary function [8], increased hospitalization risk (6.5% higher) [12], accelerated disease progression, and reduced time to transplantation [13,14]. These robust associations establish chronic cough as both a prognostic marker and therapeutic priority in ILD management [9,15].

The primary goal of this study was to evaluate the current understanding of (1) ILD subtype-specific cough prevalence, (2) HRQoL impacts, (3) pathogenic mechanisms, and (4) therapeutic evidence—aiming to optimize management strategies for this understudied complication.

This review builds on the existing literature by providing an updated, comprehensive synthesis of the mechanisms and management of chronic cough across ILD subtypes. While most previous work has focused predominantly on IPF, we systematically integrate emerging data on understudied ILDs (for example, HP, sarcoidosis, and systemic disease-associated ILDs) and evaluate recent therapeutic advances (P2X3 antagonists). Our analysis expands current understanding by characterizing subtype-specific cough phenotypes and their distinct prognostic implications—providing clinicians with a more nuanced approach to cough management in ILDs.

## 2. Materials and Methods

### 2.1. Search Strategy

We conducted a systematic search of studies published in electronic databases (PubMed, the Cochrane Library, MEDLINE, Embase). To ensure comprehensive retrieval, the authors perform the search using the keywords “chronic cough”, “interstitial lung disease”, “idiopathic pulmonary fibrosis”, “hypersensitivity pneumonitis”, “sarcoidosis”, “health-related quality of life”, “pathogenesis”, and “treatment”. The keywords were combined with Boolean operators “AND” and “OR”. In addition, the authors analyzed the bibliography of published trials, meta-analyses, and reviews for additional references. Two authors reviewed the literature and cross-checked the review.

### 2.2. Study Selection

Two authors independently screened the titles and abstracts to identify eligible studies before reading the full-text articles. Any differences in decisions about data selection or interpretation were resolved through consensus discussion. If agreement could not be reached, a third advanced author made the final decision. Study selection was limited to randomized controlled trials, observational studies (both prospective and retrospective), systematic reviews, and meta-analyses published between the late 2000s and 2024, excluding all other study designs. Inclusion criteria were studies conducted in adult patients (≥18 years) with diagnosed ILDs (IPF and non-IPF idiopathic interstitial pneumonias, autoimmune-related ILDs, exposure-related conditions, and HP) who reported chronic cough (≥8 week duration). The references of the identified studies were also manually reviewed to identify any additional relevant research that matched the specified selection criteria. Eligible studies were required to provide cough-specific outcome data, including prevalence, severity measures, HRQoL impact, or treatment efficacy. The study quality of non-randomized studies was assessed using the Newcastle–Ottawa scale. Case reports, review articles, studies of non-ILD populations, and publications lacking cough-specific outcomes were excluded. Commentaries, letters, case reports and case series, and conference abstracts were excluded to maintain methodological rigor. Also, the exclusion criteria were studies that enrolled patients without ILDs or without cough symptoms and publications lacking cough-specific outcomes. Only English-language publications were selected for final inclusion.

## 3. Results

### 3.1. Pathogenesis of Cough in ILDs

**Figure 1:**
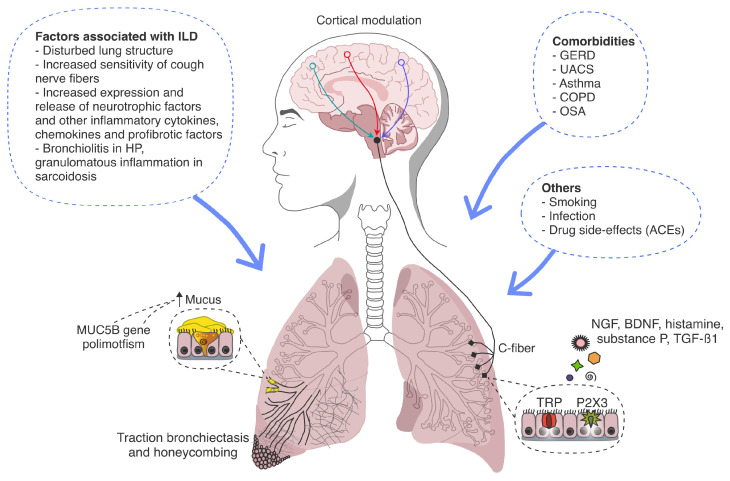
Pathogenesis of cough in ILDs. ILD, interstitial lung disease; HP, hypersensitivity pneumonitis; GERD, gastroesophageal reflux disease; UACS, upper airway cough syndrome; COPD, chronic obstructive pulmonary disease; OSA, obstructive sleep apnea; ACEs, angiotensin-converting enzyme inhibitors; NGF, neurotrophin nerve growth factor; BDNF, brain-derived neurotrophic factor; TGF-ß1, transforming growth factor β-1; TRP, transient receptor potential; P2X3, purinergic 2X3 receptor. Chronic cough in ILDs can be caused by ILD-related factors, comorbidities, and other factors. ILD-related factors include architectural distortion, cough hypersensitivity syndrome, and increased production of inflammatory and profibrotic factors that activate cough through receptors (TRP and P2X3) and C-fibers. Disease-specific mechanisms include bronchiolitis in HP and granulomatous inflammation in sarcoidosis. The MUC5B gene polymorphism, which increases bronchial mucus production, is also noted.

A wide range of pathologic pathways causing cough in patients with various ILDs are considered. Figure 1 summarizes the key pathways in the pathogenesis of cough in ILDs.

#### 3.1.1. Cough Hypersensitivity Syndrome

Mann et al. showed that cough triggers in ILD patients are diverse and include aerosol exposure, smells and fragrances, lower respiratory tract infection, temperature and humidity changes, poor air quality, windy weather, certain foods, and talking and laughing [16]. Hirons et al. found that the common triggers of cough were change in body position (74%), physical activity (72%), and talking (62%). Triggers and sensations strongly correlated with the number of impacts (*p* = 0.73, *p* < 0.001) [17].

Architectonical distortion of the lung tissue associated with the development of fibrosis affects nerve fibers and quickly adapting and slowly adapting receptors (the afferent part of the cough reflex), with increasing receptor sensitivity due to the traction effect [18]. Activity of neurons inhibiting the cough reflex can also be reduced due to progressive fibrosis [19]. The role of transforming growth factor ß-1 (TGF-ß1) in cough occurrence in patients with pulmonary fibrosis is also widely discussed [20]. Moreover, the presence of polymorphonuclears and mast cells in bronchoalveolar lavage fluid (BALF) from patients with IPF may contribute to cough, as these cells produce mediators and enzymes (histamine, serotonin, tryptase, and substance P) that activate C-fibers [21,22]. Harrison et al. also demonstrated higher levels of nerve growth factor (NGF) and brain-derived neurotrophic factor (BDNF) in the airway secretions of patients with IPF [22].

Schertel et al. demonstrated a reduction in cough severity during sleep in patients with IPF. According to the results of 24 h respiratory polygraphy in eight patients with IPF treated with antifibrotic agents, cough predominated in waking hours (mean, 14.8 (10.9–6.8)/h during wake hours vs. 1.6 (1.3–2.8)/h during sleep, *p* = 0.004) [23]. Thus, cough occurrence could be associated with cortical activity.

#### 3.1.2. MUC5B Gene Polymorphism

Mucin production and clearance could potentially influence the severity of cough in patients with ILDs. The occurrence of IPF and other ILDs is associated with MUC5B gene polymorphism, which leads to increased expression of this gene and, presumably, to a change in the production and clearance of mucus that could, in turn, increase cough [24]. Increased expression of MUC5B in the distal airways is not seen in fibrotic NSIP, suggesting that this mechanism is more specific to IPF [25]. The PROFILE study assessed the relationship between cough severity, measured by the Leicester Cough Questionnaire (LCQ), and the MUC5B promoter polymorphism. There was no significant association between LCQ scores and MUC5B genotype. This suggests that the presence of the MUC5B polymorphism does not influence cough severity in patients with IPF [26].

#### 3.1.3. Gastroesophageal Reflux

Madison et al. showed that cough was associated with ILDs in only 46.1% of cases, whereas the other causes of cough were asthma, postnasal drip syndrome, gastroesophageal reflux disease (GERD), bronchiectasis, or exacerbation of chronic obstructive pulmonary disease (COPD). The authors emphasized the need for timely diagnosis of comorbidities that could significantly contribute to the burden of respiratory symptoms and require proper treatment [27].

GERD is a common condition in people with IPF. According to a population study of IPF, the incidence of GERD was higher compared to controls (43.4% vs. 28.5%; *p* = 0.006), and the probability of having GERD was 1.78 times higher (95% CI 1.09–2.91; *p* = 0.02) in the ILD group compared to the controls [28,29]. Cough is one of the most common extraesophageal manifestations of GERD and can be caused by the direct exposure of gastric content to cough receptors in the larynx and the upper airways [30].

#### 3.1.4. Disease-Specific Mechanisms

Mechanisms of cough in sarcoidosis are slightly different than in other ILDs and are primarily related to a combination of parenchymal injury with airway deformities and granulomatous inflammation associated with airflow limitation, bronchial hyperreactivity, and probably cough hypersensitivity syndrome [6,31]. According to the findings of Xin-Min et al., cough was significantly more common in patients with sarcoid lesions of the tracheal mucosa compared to patients with sarcoidosis without trachea injury (92.3% vs. 49.2%, *p* < 0.001) [32]. BALF concentrations of NGF and neurotrophin-3 (NT-3) were significantly increased, and immunoreactivity against NGF, BDNF, NT-3, and tyrosine protein kinase receptors (TrkA, TrkB, and TrkC) in granulomas was found in patients with sarcoidosis using immunohistochemical analysis [33]. It is known that neurotrophins promote differentiation and survival of neurons in the central and the peripheral nervous systems by increasing the sensitivity of afferent neurons in the lungs and activating cough.

The pathogenesis of cough in HP is poorly understood, but the role of airflow limitation due to bronchiolitis is discussed, and some cough mechanisms described in IPF may also play a role in fibrotic HP [10].

### 3.2. Clinical Importance of Cough in ILDs

There are different ways to assess chronic cough, both subjectively and objectively. The visual analog scale (VAS) is a subjective tool. A strong correlation was shown between objective measures of cough incidence and VAS score (in wake, r = 0.80; *p* < 0.001; during sleep, r = 0.71; *p* = 0.001) [34]. It should also be mentioned that the rate of coughing during the day was much higher than during the night (median, 14.6; range 1.9–56.6, compared to 1.9; range 0–19.2; *p* = 0.003) [34]. Cheng et al. showed that cough intensity in patients with IPF or chronic HP was significantly higher than in SS-ILD (29 (11–48), 29 (11–48), and 18 (0–33) mm of VAS, respectively, *p* < 0.0001). Productive cough was more common in patients with chronic HP than in patients with IPF or SS-ILD (63% compared to 43% and 21%, respectively, *p* < 0.001). In this study, cough was associated with a lower diffusing capacity of the lungs (DLco) (*p* = 0.03) and was not related to comorbidities or the patient’s treatment [9]. A comparative analysis of cough in ILDs was also carried out by Sato et al., who included patients with IIPs (*n* = 70), CTD-associated ILDs (*n* = 49), and chronic HP (*n* = 10). Patients with IIPs had the highest intensity of cough, measured by VAS (31 vs. 24 in CTD-associated ILDs and 18 in chronic HP, *p* = 0.048). The intensity and frequency of cough were inversely related to DLco and were directly related to the composite physiological index (CPI). Additionally, researchers showed a relationship between cough and clinical manifestations of GERD; this fact should be considered when selecting the therapy [8].

Yamamura et al. showed that 6 of 23 patients with IPF (26.1%) experienced refractory cough. Among these patients, 83.3% exhibited traction bronchiectasis and distorted airway architecture on computer tomography (CT) scans, compared to only 11.8% of IPF patients without cough (*p* < 0.01). Architectural abnormalities, such as honeycombing and airway distortion, can increase mechanical stress on the airways, potentially heightening cough sensitivity by activation of sensory nerves like Aδ-fibers [35].

#### 3.2.1. Health-Related Quality of Life

Cough significantly affects HRQoL in patients with ILDs, worsening both physical and psychological health [16,36]. The consequences of chronic cough in adults include urinary incontinence, chest pain, speech impairment, anxiety and depression, social limitations, and disability [37]. According to Cheng et al., cough is described as painful by 27% of patients with ILDs; fatigue associated with cough was determined in 38%, sleep disturbance in 39%, and discomfort in public space in 34% of patients [9]. The severity of cough in patients with ILDs is an independent predictor of decreased HRQoL according to the St. George Respiratory Questionnaire (SGRQ), adjusted for age, gender, ILD severity, and dyspnea (*p* = 0.03) [9].

Green et al. found that patients with IPF experience significant HRQoL reductions, as reflected by Cough and Sputum Assessment Questionnaire and SGRQ scores (287.90 and 57.47, respectively), indicating cough-related impairments. Cough disrupts daily life, causing exhaustion and sleep disturbances, particularly during exertion. Additionally, cough is more prevalent and severe in IPF than in other ILDs or COPD, contributing to a greater HRQoL burden [38].

Several questionnaires, such as the LCQ or the cough-specific questionnaire (CQLQ), are self-completed HRQoL measures of chronic cough that are often used to describe the efficacy of cough therapy in ILDs [39].

#### 3.2.2. Progression of ILDs

Zaman et al. showed that cough was associated with worse transplant-free survival in men with IPF (OR, 1.40; 95% CI 1.14–1.72; *p* = 0.007), but not in women. The authors suggest that this difference could be due to different causes of cough in men and women. For example, men more often reported a history of smoking, whereas cough in women was more often associated with heartburn [14].

According to Ryerson et al., cough was associated with an increased risk of IPF progression (OR, 4.97; 95% CI 1.25–19.80; *p* = 0.02), while the association of cough with mortality did not reach the statistical significance [15]. Viet et al. compared cough measured by VAS in patients with IPF and other fibrotic ILDs. They showed that 94.3% of the patients complained of cough, while the severity of cough and cough progression within 6 months were higher in patients with IPF (*p* = 0.020 and *p* = 0.009, respectively). Also, patients who died or underwent lung transplantation had a higher VAS score for cough (*p* = 0.047). Cough severity was a significant predictor of transplant-free survival (HR, 1.387; 95% CI 1.081–1.781; *p* = 0.010) [40].

Lee et al. showed that a reduction in cough-related QoL measured by the LCQ was associated with an increased risk of hospitalization for respiratory diseases (HR, 1.065; 95% CI 1.025–1.107), death (HR, 1.074; 95% CI 1.020–1.130), and the need for lung transplantation (HR, 1.087; 95% CI 1.022–1.156) within one year in 1447 patients with different ILDs selected from a registry [12].

Two clinical studies on SS-ILD have shown that more severe cough in these patients was associated with more severe pulmonary disease [41,42]. Theodore et al. showed that patients with cough had lower DLco, more prominent dyspnea, and more extended pulmonary fibrosis on chest HRCT compared to patients without cough. The intensity of cough was also negatively related to the forced vital capacity (FVC) [41]. Tashkin et al. reported that patients with SS-ILD and chronic cough (61.3% of patients) had more severe dyspnea, more prominent interstitial lesions on chest HRCT, and lower DLco. The authors recommended considering cough as an additional marker of treatment efficacy in clinical studies of SS-ILD [42].

Nagy et al. analyzed predictors of SS-ILD progression in patients with normal baseline FVC (> 80% of predicted). Chronic cough and pulmonary hypertension were found to be the key predictors of progression of ILD (OR, 36.2 (95% CI 1.8–711.9) and 36.4 (95% CI 1.1–1184.9), respectively) [43].

### 3.3. Treatment of Cough in ILDs

ILD patients with cough are most commonly prescribed anti-reflux drugs, inhaled bronchodilators, inhaled corticosteroids (ICSs), and opiates, while antitussives, intranasal corticosteroids, and antihistamines are the least effective (Table 2) [44,45].

#### 3.3.1. Antifibrotic Therapy

The mainstay of IPF treatment is antifibrotic drugs such as pirfenidone and nintedanib. In the study of van Manen et al., 12-week therapy with pirfenidone resulted in the improvement in cough by 34% in 20 of 27 patients with IPF (74%), according to 24 h cough monitoring. Subjective VAS scores also improved. These results demonstrated the potential of pirfenidone to treat cough in ILDs patients, but this effect needs more detailed evaluation [46]. In the study by Azuma et al., pirfenidone reduced the decline in vital capacity (VC) as well as cough and dyspnea severity in a subgroup of patients with IPF and VC, (% predicted) > 70% and SpO2 < 90% at baseline [47]. A recent systematic review and meta-analysis showed that patients with IPF and advanced pulmonary fibrosis treated with nintedanib had a lower risk of developing cough (12.1% vs. 15.6% (OR, 0.73; 95% CI 0.56–0.96; *p* = 0.002) and dyspnea (8.9% vs. 12.5% (OR, 0.70; 95% CI 0.53–0.94; *p* = 0.02) [48]. Wijsenbeek et al. demonstrated significantly less progression of dyspnea, fatigue, and cough, as measured by the Living with Pulmonary Fibrosis (L-PF) questionnaire, in patients with progressive fibrosing ILDs (excluding IPF) treated with nintedanib for 52 weeks compared with the placebo group [49].

#### 3.3.2. Inhaled Therapy

A pilot study of inhaled cromolyn sodium in patients with IPF showed promising results, reducing cough frequency by > 30% compared to the placebo group. Cromolyn sodium was well tolerated, but no change in objective data was found due to the short duration of the study [50]. However, Martinez et al. in a multicenter, randomized, placebo-controlled study showed no positive effect of cromolyn sodium on cough severity compared with placebo in patients with IPF, regardless of the dosage used (10 mg, 40 mg or 80 mg t.i.d.) (*p* = 0.36, *p* = 0.27, and *p* = 0.95, respectively) [51].

ICSs are also considered a therapeutic option. Several studies showed no significant clinical effect of ICSs on chronic cough [52,53,54], whereas Pietinalho et al. reported a clinical improvement in cough with inhaled budesonide 800 mcg daily following a course of systemic steroids [55].

A pilot study of highly selective inhaled carcainium chloride (a quaternary derivative of lidocaine) versus placebo in patients with IIP showed a significant reduction in cough frequency (*p* < 0.001) and cough severity VAS scores (*p* < 0.001) in the main group versus placebo [70].

#### 3.3.3. Immunomodulating Therapy

A reduction in cough severity was demonstrated in a small non-randomized study after treatment with oral corticosteroids in IPF, but this therapy did not improve HRQoL and survival of the patients. Moreover, side effects of systemic steroids limited their use [56,71]. The use of systemic corticosteroids for cough in IPF could be considered in patients with exacerbation of IPF, asthma, or eosinophilic bronchitis [72].

Theodore et al. presented a randomized controlled trial in which cough associated with SS-ILD was successfully treated with oral cyclophosphamide for one year. However, the treatment effect was relatively small and disappeared after the treatment was stopped [41]. Tashkin et al. also found that patient-reported cough reduced by 41% and 44% in patients with SS-ILD treated with mycophenolate mofetil for two years or with cyclophosphamide for one year, respectively, but no one drug improved cough-associated QoL measured by the LCQ [42].

Considering the immunomodulatory and anti-inflammatory activity of azithromycin and positive experience of its use in COPD and idiopathic chronic cough [73,74], Guler et al. conducted a study investigating the effect of azithromycin on the severity and frequency of cough in patients with IPF [57]. Unfortunately, cough was not improved under this therapy according to the LCQ, VAS, and polygraphy.

Another drug, thalidomide, improved cough and QoL in patients with IPF in a 24-week single-center, double-blind cross-over study due to its anti-inflammatory and antiangiogenic effects [58]. At the same time, thalidomide also has multiple side effects including dizziness, constipation, and serious adverse events such as thromboembolism and peripheral neuropathy [75,76].

#### 3.3.4. Anti-Reflux Therapy

In a recent randomized double-blind study by Dutta et al., there was no significant difference in disease progression and respiratory function decline between patients with IPF treated with omeprazole or placebo, but a 39% reduction in cough frequency was noted under the treatment with omeprazole [59]. Therefore, therapy with proton pump inhibitors (PPIs) for cough in patients with IPF without confirmed GERD and negative pH monitoring is not recommended [72].

#### 3.3.5. Neuromodulating Therapy

Increased sensitivity of airway afferent neurons is the most likely mechanism of cough in fibrotic ILDs. Therefore, in cases where cough is related to ILDs and cough-related comorbidity is either excluded or successfully treated, use of neuromodulators seems rational [76]. A systematic review of neuromodulators for idiopathic chronic cough showed that use of gabapentin, pregabalin, amitriptyline, or baclofen could decrease cough intensity [61]. In a randomized controlled trial, gabapentin was shown to significantly improve QoL as measured by the LCQ in patients with chronic cough compared with placebo, although 31% of patients experienced adverse events (most commonly generalized weakness and nausea) [62]. These agents appear effective for refractory chronic cough, though specific data for ILDs are lacking.

#### 3.3.6. Novel Antitussive Agents

Recently, a promising effect of a novel antitussive agent, the P2X3 receptor antagonist gefapixant, on unexplained chronic cough or refractory cough has been demonstrated in double-blind, randomized, parallel-group, placebo-controlled, phase III trials COUGH1 and COUGH2 [63]. P2X3 is expressed by vagal airway afferent nerves and contributes to the hypersensitivity of sensory neurons [77].

In the COUGH-2 trial, participants receiving gefapixant 45 mg had significantly higher odds of achieving a ≥1.3-point improvement in the LCQ total score compared to placebo (OR 1.41, 95% CI 1.02–1.96; *p* = 0.040). A reduction in cough severity was achieved due to controlling the afferent sensitivity of the upper and lower airways by blocking P2X3 receptors, which are ion channels located in afferent neurons. It is expected that modulation of cough neural pathways would also be effective in patients with ILDs and chronic cough [63].

A multicenter, randomized, double-blind, phase III trial conducted in Japan evaluated the efficacy of gefapixant at doses of 15 mg and 45 mg in patients with refractory or unexplained chronic cough (*n* = 169). Both treatment groups showed improvement in cough-related quality of life, as assessed by the LCQ. At Week 12, a clinically significant increase (≥1.3 points) in the total LCQ score was observed in 48% of patients receiving 15 mg gefapixant and 41% of those receiving 45 mg. By the end of the study (Week 52), these rates increased to 59% and 47%, respectively [78]. However, in a randomized, placebo-controlled study by Martinez et al., gefapixant 50 mg daily did not significantly reduce cough in patients with IPF compared with placebo (*p* = 0.90) [64].

#### 3.3.7. Opiates

Opiates are recommended as palliative care in patients with advanced fibrosis and troublesome cough [72]. Low-dose slowly released morphine (5 to 10 mg twice daily) was shown to significantly improve cough-related QoL and to reduce cough intensity by 40% during the first 5 days of therapy in patients with chronic cough [65]. A multicenter, double-blind, placebo-controlled cross-over study of morphine sulfate in patients with IPF-induced cough (NCT04429516) is currently underway. In a randomized clinical trial PACIFY COUGH, morphine (5 mg orally twice a day for 14 days) reduced the number of coughs per hour, as well as awake cough frequency by 39.4% compared with placebo [66]. Morphine can be used to treat severe cough in patients with advanced pulmonary fibrosis, but it can also reduce the protective function of the cough reflex and can adversely affect the respiratory center, which prevent the use of opioids in such patients [71]. Nalbuphine belongs to the opioid agonist–antagonist class of drugs. Oral nalbuphine in an extended-release form has the potential to provide the therapeutic benefits of opioid medications while minimizing side effects. For this reason, in a randomized, double-blind study by Maher et al., 41 participants were given either nalbuphine or a placebo. The results showed a significant reduction in daytime cough frequency during the nalbuphine treatment period, with a 75.1% reduction compared to a 22.6% reduction during the placebo period. This resulted in a placebo-adjusted reduction of 52.5 percentage points (*p* < 0.001) at day 21 [67].

#### 3.3.8. Non-Pharmacological Therapy

Some experts suggested a positive effect of supplemental oxygen on the severity of cough in patients with IPF [76]. The study by Khor et al. showed an improvement in the psychological domain score of the LCQ, indicating better cough-related QoL (mean difference of 0.9; 95% CI 0.2 to 1.6; *p* = 0.01); the authors note that the results presented are a secondary surrogate endpoint [68]. However, relevant clinical studies have not yet been published.

Refractory chronic cough in patients with ILDs could be treated with speech therapy [79]. Combined treatment with pregabalin and speech therapy may result in greater reduction in cough than speech therapy alone [80]. A randomized controlled trial in 75 patients with chronic refractory cough demonstrated that a 4-week combination of physiotherapy, speech therapy, and language therapy significantly improved cough-related QoL and reduced cough intensity [69].

Physiotherapists and rehabilitation specialists also recommend the use of cough control therapy, which involves learning about the mechanics of coughing, identifying triggers, and learning various techniques to suppress coughing. Therapy also includes psychosocial support aimed at modifying behavior and improving the overall QoL for people with chronic cough [81].

## 4. Discussion

The articles reviewed in this paper highlight the main points of pathogenesis, clinical impact of, and current therapeutic approaches to chronic cough in ILDs. Cough is a frequent and debilitating symptom in patients with ILD. Patients with ILDs may experience a diverse range of clinical characteristics related to chronic cough, including a negative impact on their HRQoL [16]. Some authors consider cough as an important clinical marker of disease severity and even as a potential predictor of ILD progression [8,12,15,40].

The pathophysiological mechanisms of chronic cough in ILDs are not fully understood, but every potential mechanism could play an independent role in a certain ILD, and, therefore, could require an individual therapeutic option. A number of researchers refer to ILD-related cough as cough hypersensitivity syndrome, which is characterized by an upregulated cough reflex in response to mild nonspecific (trivial) stimuli (laughter, phonation, temperature changes, perfume, etc.) [19,82,83]. Given the advanced fibrosing process in some ILDs, architectural distortion of the lung parenchyma could be a potential cause of chronic cough [19]. Genetic predisposition to chronic cough is also discussed, including MUC5B gene polymorphism, but the findings are conflicting and require further investigation [24,25,26]. ILD-related cough could be due to the overexpression of inflammatory cytokines, neutrophils, and profibrotic factors [33].

It should be noted that various comorbidities, including GERD, asthma, COPD, obstructive sleep apnea syndrome, and lung cancer, can also contribute to chronic cough in patients with ILDs [75].

The primary goal of therapy for ILD-related chronic cough is to reduce the cough burden and its impact on HRQoL. Unfortunately, there are few effective, evidence-based therapeutic options for chronic cough in patients with ILDs.

ICSs were traditionally considered as a therapeutic strategy for cough, but the evidence of their efficacy is controversial [52,53,54]. The effect of immunosuppressive therapy on ILD-related cough was also evaluated, especially since this therapy is used in a number of treatments for ILDs themselves. Unfortunately, there was no significant effect on cough severity. Moreover, a wide range of adverse effects does not allow these agents to be used as a symptom-relief therapy [42,56,57,71,73,74].

Some studies showed that antifibrotic agents in IPF and immunosuppressive agents in SS-ILD could reduce cough, but prospective clinical trials are needed to confirm these results [42,47,48].

Low-doses opiates are often used to reduce refractory cough in patients with terminal stages of ILDs, but thorough monitoring of adverse effects should be performed. The opioid agonist–antagonist nalbuphine could also manage refractory cough without serious adverse effects [67].

Attempts have been made to use local anesthetics to inhibit peripheral sensory nerve conduction and to suppress cough; however, their use is limited due to the low selectivity of these agents, their short duration of action, and their known interference with other important protective reflexes, such as gagging and swallowing reflexes [84,85]. Due to the increased sensitivity of airway afferent neurons, the use of neuromodulators in ILD-related cough seems more reasonable [61,62].

Investigation of P2X3 receptor antagonists have given promising results. In particular, several molecules are currently under investigation and have already shown an acceptable safety profile and clinical efficacy [86].

Where co-morbid conditions exist that may be the cause of the cough, the optimal management of these conditions is recommended. The role of GERD in IPF patients with chronic cough is actively discussed, though the effects of anti-reflux therapy, including PPIs and H2-blockers, are controversial. PPIs could decrease acid reflux in IPF patients, but failed to improve both the frequency and severity of cough in patients with non-acid GERD [60].

Patients with refractory ILD-related cough should also be advised to use non- pharmacological therapeutic methods, including supplemental oxygen, speech therapy, and pulmonary rehabilitation [68,76].

This review has several limitations that reflect gaps in the existing literature. Most available studies focused specifically on IPF, with limited data on other ILD subtypes. Significant heterogeneity exists both between studies (due to different cough assessment tools and often small sample sizes) and within the study populations themselves—as the result of fundamental differences between ILD groups (for example, IPF, NSIP, and HP) and diversity between categories (fibrotic and nonfibrotic). These differences complicate pooled analysis and highlight the need for ILD subtype-specific studies. In addition, the predominance of observational studies and the lack of standardized protocols between studies may affect the generalizability of conclusions.

## 5. Future Studies

Future research on ILD-related chronic cough should focus on understanding subtype-specific underlying mechanisms and developing targeted treatments. The role of neurotrophins, inflammatory mediators, and lung architectural distortion in cough hypersensitivity requires further investigation. Additionally, the impact of MUC5B gene polymorphism on cough severity remains unclear and warrants genomic research.

Pharmacological advancements, including antifibrotics, neuromodulators, and P2X3 receptor antagonists, hold promise but need further evaluation in ILD populations. Non-pharmacological approaches such as speech therapy, pulmonary rehabilitation, and oxygen therapy should also be explored. Given the influence of comorbidities like GERD and asthma, personalized treatment strategies are essential.

Standardized cough assessment tools and digital monitoring technologies could improve clinical trials, while longitudinal studies may clarify the relationship between cough severity and ILD progression. Addressing these research gaps will enhance symptom management and quality of life for ILD patients.

## 6. Conclusions

Chronic cough in ILDs is a potentially debilitating symptom that significantly reduces the patient’s QoL and affects many other aspects of life. The pathophysiological basis and therapeutic methods for chronic cough in various ILDs require further investigations to identify targets for drug therapy. The severity of the cough may reflect the severity of ILDs, but similarly, it could be associated with some comorbidities, which should be taken into account when prescribing specific therapy to a patient. The main therapeutic approaches are the use of antifibrotics, neuromodulators, opiates, inhaled local anesthetics, oxygen therapy, speech therapy, and treatment of gastroesophageal reflux disease. New compounds, such as P2X3 antagonists, are under investigation and have great potential. Thus, a comprehensive assessment of cough is required for effective cough treatment in a patient with ILDs, considering possible mechanisms and individual impact on QoL.

## Figures and Tables

**Table 2:** Treatment of cough in ILDs.

Treatment	Effect	Reference
Pirfenidone/nintedanib	reduce cough severity in IPF and PPF decrease risk of developing cough in IPF reduce cough progression in PPF	van Manen, 2017 [46] (prospective, observational study; *n* = 43) Azuma, 2011 [47] (double-blind, randomized, placebo-controlled trial; *n* = 275) Chen, 2021 [48] (systematic review and meta-analysis of randomized controlled trials; *n* = 2583) Wijsenbeek, 2024 [49] (double-blind, randomized, placebo-controlled trial; *n* = 663)
Inhaled cromolyn sodium	reduce cough frequency in IPF no effect was found for IPF	Biring, 2017 [50] (double-blind, randomized, placebo-controlled trial; *n* = 24) Martinez, 2021 [51] (double-blind, randomized, placebo-controlled trial; *n* = 108)
Inhaled corticosteroids	no effect was found for sarcoidosis decrease in cough severity in sarcoidosis (inhaled budesonide + systemic steroids)	Milman, 1994 [52] (double-blind, randomized, placebo-controlled trial; *n* = 21) du Bois, 1999 [53] (double-blind, randomized, placebo-controlled trial; *n* = 44) Baughman, 2002 [54] (double-blind, randomized, placebo-controlled trial; *n* = 21) Pietinalho, 1999 [55] (double-blind, randomized, placebo-controlled trial; *n* = 189)
Oral corticosteroids	decrease in cough severity in IPF but lead to side effects	Hope-Gill, 2003 [56] (non-randomized intervention clinical trial; *n* = 20)
Oral cyclophosphamide	decrease in cough severity in SS-ILD was relatively small	Theodore, 2012 [41] (double-blind, randomized, placebo-controlled trial; *n* = 156)
Mycophenolate mofetil	decrease in cough severity in SS-ILD	Tashkin, 2017 [42] (randomized, placebo-controlled trial; *n* = 142)
Azithromycin	no effect was found in IPF	Guler, 2021 [57] (randomized, placebo-controlled crossover trial; *n* = 25)
Thalidomide	decreases in cough severity in IPF but leads to side effects	Horton, 2012 [58] (randomized, placebo-controlled crossover trial; *n* = 23)
Proton pump inhibitors	decrease in cough severity and frequency in IPF	Dutta, 2019 [59] (randomized, placebo-controlled crossover trial; *n* = 45) Kilduff, 2014 [60] (non-randomized intervention clinical trial; *n* = 18)
Neuromodulators (gabapentin, pregabalin, amitriptyline, baclofen)	decrease in cough severity in IPF but lead to side effects	Cohen, 2013 [61] (systematic review; *n* = 14) Ryan, 2012 [62] (double-blind, randomized, placebo-controlled trial; *n* = 62)
P2X3 receptor antagonist (gefapixant)	decrease in cough severity and frequency in ILDs no effect was found in IPF	McGarvey, 2022 [63] (double-blind, randomized, placebo-controlled trial; *n* = 2049) Martinez, 2021 [64] (double-blind, randomized, placebo-controlled crossover trial; *n* = 51)
Opiates (morphine) and the opioid agonist-antagonist (nalbuphine)	opiates decrease cough severity and frequency in patients with chronic cough but lead to side effects opioid agonist–antagonist decreased daytime cough frequency	Morice, 2007 [65] (double-blind, randomized, placebo-controlled trial; *n* = 27) Wu, 2024 [66] (double-blind, randomized, placebo-controlled crossover trial; *n* = 44) Maher, 2023 [67] (double-blind, randomized, placebo-controlled crossover trial; *n* = 41)
Supplemental oxygen	improvement in the LCQ, indicating better cough-related QoL	Khor, 2020 [68] (triple-blind, randomized, placebo-controlled trial; *n* = 30)
Speech therapy	decrease in cough severity and frequency	Chamberlain, 2017 [69] (randomized, placebo-controlled trial; *n* = 75)

ILDs, interstitial lung diseases; IPF, idiopathic pulmonary fibrosis; PPF, progressive pulmonary fibrosis; SS-ILD, systemic sclerosis-associated interstitial lung disease; QoL, quality of life; LCQ, the Leicester Cough Questionnaire.

## Data Availability

No new data were created or analyzed in this study.
